# The Critical Role of Arbuscular Mycorrhizal Fungi to Improve Drought Tolerance and Nitrogen Use Efficiency in Crops

**DOI:** 10.3389/fpls.2022.919166

**Published:** 2022-07-06

**Authors:** Haiying Tang, Muhammad Umair Hassan, Liang Feng, Muhammad Nawaz, Adnan Noor Shah, Sameer H. Qari, Ying Liu, Jianqun Miao

**Affiliations:** ^1^College of Agriculture and Biotechnology, Hunan University of Humanities, Science and Technology, Loudi, China; ^2^Research Center on Ecological Sciences, Jiangxi Agricultural University, Nanchang, China; ^3^College of Agronomy, Sichuan Agricultural University, Chengdu, China; ^4^Sichuan Engineering Research Center for Crop Strip Intercropping System, Key Laboratory of Crop Eco-physiology and Farming System in Southwest, Ministry of Agriculture and Rural Affairs, Chengdu, China; ^5^Department of Agricultural Engineering, Khwaja Fareed University of Engineering and Information Technology, Rahim Yar Khan, Pakistan; ^6^Department of Biology, Al-Jumum University College, Umm Al-Qura University, Makkah, Saudi Arabia; ^7^School of Computer Information and Engineering, Jiangxi Agricultural University, Nanchang, China

**Keywords:** AMF, antioxidant defense system, aquaporins, drought stress, genes expression, hormones, NUE

## Abstract

Drought stress (DS) is a serious abiotic stress and a major concern across the globe as its intensity is continuously climbing. Therefore, it is direly needed to develop new management strategies to mitigate the adverse effects of DS to ensure better crop productivity and food security. The use of arbuscular mycorrhizal fungi (AMF) has emerged as an important approach in recent years to improve crop productivity under DS conditions. AMF establishes a relationship with 80% of land plants and it induces pronounced impacts on plant growth and provides protection to plants from abiotic stress. Drought stress significantly reduces plant growth and development by inducing oxidative stress, disturbing membrane integrity, plant water relations, nutrient uptake, photosynthetic activity, photosynthetic apparatus, and anti-oxidant activities. However, AMF can significantly improve the plant tolerance against DS. AMF maintains membrane integrity, improves plant water contents, nutrient and water uptake, and water use efficiency (WUE) therefore, improve the plant growth under DS. Moreover, AMF also protects the photosynthetic apparatus from drought-induced oxidative stress and improves photosynthetic efficiency, osmolytes, phenols and hormone accumulation, and reduces the accumulation of reactive oxygen species (ROS) by increasing anti-oxidant activities and gene expression which provide the tolerance to plants against DS. Therefore, it is imperative to understand the role of AMF in plants grown under DS. This review presented the different functions of AMF in different responses of plants under DS. We have provided a detailed picture of the different mechanisms mediated by AMF to induce drought tolerance in plants. Moreover, we also identified the potential research gaps that must be fulfilled for a promising future for AMF. Lastly, nitrogen (N) is an important nutrient needed for plant growth and development, however, the efficiency of applied N fertilizers is quite low. Therefore, we also present the information on how AMF improves N uptake and nitrogen use efficiency (NUE) in plants.

## Introduction

Drought stress (DS) is a serious abiotic stress, negatively affecting plant growth and development across the globe (Amiri et al., 2015; Hassan et al., 2020). The recent increase in climate change has increased the intensity of DS which is posing a serious challenge to global food security (Behrooz et al., 2019; Huang et al., 2020). Drought stress is the greatest threat to field crops and it has a direct impact on crop yields and the global economy (Dolan et al., 2021; He et al., 2022). Drought stress negatively affects the plant process ranging from seed germination, growth, and final productivity. Seed germination is an essential process in the growth of plants (El-Badri et al., 2021). Successful crop productivity mainly depends on seed germination and early plant growth has direct linking with seeds’ ability to sprout under DS (Khan et al., 2019). Drought stress severely affects the activities of hormones, mobilization of stored materials, and protein structure that negatively affect the seed germination and subsequent growth of plants (Abdel-Ghani et al., 2015; Basal et al., 2020). DS also affects enzymatic activities and reduces nutrient assimilation and nutrient uptake resulting in huge yield losses (Ahanger and Agarwal, 2017). Moreover, DS also impairs the photosynthetic process, and plant water contents and reduces the synthesis of photosynthetic pigments which negatively affects plant growth (Hellal et al., 2018; Mamnabi et al., 2020). Besides this, DS also impairs the structural integrity of photosynthetic apparatus which is the main reason for reduced growth under DS (Zhang et al., 2015).

Drought stress also reduces photosynthesis by reducing cell turgor and plants’ access to CO_2_ owing to the closure of stomata (Jaleel et al., 2007). One of the major detriments of DS is the production of reactive oxygen species (ROS) that damage the structural integrity of membranes, proteins, and DNA (Ahanger and Agarwal, 2017; Sultan et al., 2021; Qari et al., 2022; Rehman et al., 2022). However, plants have developed different mechanisms to reduce the harmful effects of ROS to protect the stability of cellular structures and improve the yield under DS (Ahanger and Ahmad, 2019). The key mechanisms developed by plants to mitigate the adverse impacts of DS are better accretion of osmolytes and secondary metabolites and activation of the anti-oxidant defense system (Ahmad, 2010; Amiri et al., 2015).

Arbuscular mycorrhizal fungi (AMF) is one of the most distributed fungi across the globe that forms symbiosis association with more than 80% of terrestrial plant species (Behrooz et al., 2019). The symbiosis relationship formed between plants and AMF is beneficial for plant growth, nutrient uptake, soil quality, and stress resistance (Bi et al., 2019; Ahmed et al., 2020; Gupta, 2020; Hu et al., 2022). The symbiosis relationship between the host plant and AMF substantially improved the resistance to drought stress (Hashem et al., 2019; Zhang et al., 2019). The regulation of DS in plants by AMF is a complex process that involves diverse metabolic pathways and metabolites (Aalipour et al., 2020; Huang et al., 2020). AMF improves the survival of seedlings (Wu and Zou, 2017), and promote water uptake and transportation in the host plant (Quiroga et al., 2019a,b; Ren et al., 2019; Ortas et al., 2021), improve plant water use efficiency and gas change abilities (Quiroga et al., 2019a; Huang et al., 2020), change the morphology of roots (Quiroga et al., 2019b; Zhang et al., 2019), regulate hormone levels (Begum et al., 2020; Rydlová and Püschel, 2020), and reduce the production of ROS (Amiri et al., 2015) and thereby reduce the adverse impacts of DS. Additionally, AMF also produces glomalin, which is also known as glomalin-related soil protein (GRSP), which works as a glue that promotes the formation of water-stable aggregates by physical entanglement of extraradical hyphae, thus improving the soil water holding capacity and stabilization of soil structure (Santander et al., 2017; Gupta, 2020). Additionally, AMF also up-regulates anti-oxidant activities, osmolytes accumulation, gene expression and maintains the plant water status and photosynthetic performance under DS and resulting in a significant increase in DS tolerance (Al-Arjani et al., 2020; Seutra et al., 2021; Wang et al., 2022).

Nitrogen (N) is an essential nutrient needed for plant growth (Nishida and Suzaki, 2018). However, the efficiency of applied nitrogenous fertilizers to fulfill the plant needs is quite low (40–45%) which needs to be improved for reducing the impact on the environment (Chien et al., 2016). The excessive use of N fertilizers increases the emissions of greenhouse gases (GHGs) particular nitrous oxide (N_2_O) which is serious concern across the globe (Abeydeera et al., 2019). Globally, different efforts are being made to improve the nitrogen use efficiency (NUE) in plants. Among these efforts the use of microbes has emerged as an excellent strategy to improve NUE. Among different microbes AMF can significant improve the efficiency of N and other nutrients in field crops by increasing the surface area of roots to ensure the better absorption of nutrients (Tajini et al., 2011). AMF induce NO_3_^–^ and NH_4_^+^ transporters in plants, therefore, effect N uptake in plants (Koegel et al., 2013). AMF also bring changes in compositions of soil microbial communities by changing the development of denitrifying, nitrifying and diazotrophic symbiotic or free-living bacteria which in turn affect the N uptake and subsequent N availability to plants (Veresoglou et al., 2011). In this review, we have presented detailed information about the role of AMF in inducing the DS tolerance in plants. We have systematically presented different mechanisms of AMF mediated DS tolerance in plants. We have also discussed the research gaps that need to be filled in future studies for improving the crop production under DS with AMF. Additionally, we have also discussed the role of AMF in improving NUE in plants for ensuring better growth of plants.

## Plant Responses to Drought Stress

**FIGURE 1:**
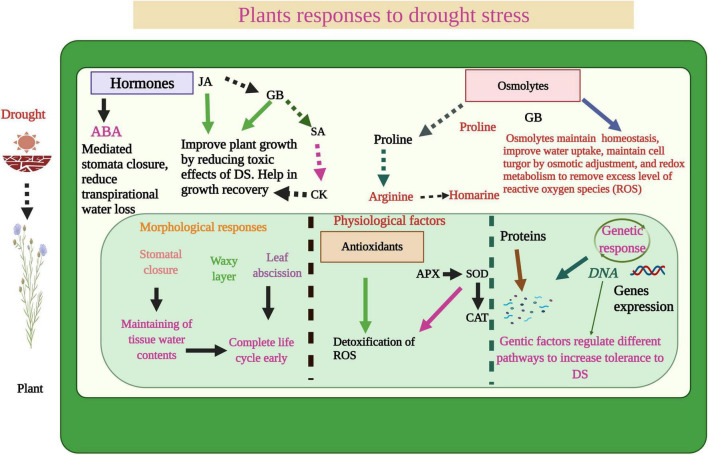
Plant responses to DS. The water deficiency disturbs plant physiological processes, plant photosynthetic efficiency, induces stomata closure, and ROS production which significant reduction in plant growth. However, plants activate an antioxidant defense system, accumulate different osmolytes, increase gene expression, produce a waxy layer and complete the early life cycle to mitigate the adverse impacts of DS.

Drought stress affects each stage of plant growth, however, germination is a very crucial stage of plant life. Drought reduces seed germination and leads to poor seedling growth and development (Khan et al., 2019). Water deficiency reduces seed germination owing (Figure 1) to a reduction in water uptake and availability of stored food, and inactivation of enzymes involved in the germination process (Jabbari et al., 2013). Drought also causes a significant decrease in plant growth by decreasing the cell expansion, increasing leaf shedding, and impairing the processes of mitosis (Liu et al., 2013; Yang et al., 2021; Batool et al., 2022a,b). DS also brings many morphological changes in plants, likewise, it reduces leaf area, leaf size, and root and shoot growth owing to activation of abscisic acid (ABA) precursor (ACC) which prevents root growth (Hewedy et al., 2021; Sayer et al., 2021). Despite this the activation of the ABA precursor also induces early maturity, leaf rolling and folding, and stomata closing which negatively affect the photosynthetic process and subsequent growth and development (Hewedy et al., 2021; Sayer et al., 2021). Moreover, DS also reduces the nodule growth and their functioning which in turn reduces the N fixation and leads to a significant decrease in the growth and production of legume crops (Furlan et al., 2012; Wilmowicz et al., 2020).

Photosynthesis is the main process negatively affected by DS. The decrease in the photosynthetic process under DS occurs due to stomatal and non-stomatal limitations (Bogati and Walczak, 2022). The stomata limitations are considered to be the main reason for decrease in the photosynthetic rate under mild DS, whereas non-stomata limitations are the main reason for reduction in photosynthetic rate under severe DS (Bogati and Walczak, 2022). Stomata closing limits the carbon dioxide (CO_2_) absorption and prevents transpiration losses owing to reduced water potential (Yang et al., 2021). In the case of non-stomata factors, the reduced activity of RuBisCO and reduction in efficiency of PS-II substantially reduce the photosynthetic rate under DS (Ma et al., 2015).

Drought also induces various biochemical changes in plants (Table 1). Likewise, DS reduces the synthesis of chlorophyll contents and increases the proline contents, and causes oxidative damage by inducing the production of ROS (Hassan et al., 2017). Reactive oxygen species (ROS) produced by DS synthesis of chlorophyll and photosynthetic apparatus and lead to a serious reduction in photosynthesis and subsequent assimilate production (Bogati and Walczak, 2022). Drought stress also disturbs electron transport and decreases the pool size of electron acceptors (Feng and Cao, 2005; Azzeme et al., 2016), also leading to marked reduction in photosynthesis. Drought-induced ROS also damage membrane integrity and cause oxidation of proteins, DNA, nucleic acid, lipids, and carbohydrates (Sabra et al., 2012; Dossa et al., 2017; Hassan et al., 2019, 2021; Bao et al., 2020; Jinhu et al., 2022; Khan et al., 2022a,b). However, plants accumulate different osmolytes to counter the effects of DS. Among different osmolytes, proline (Pro) is an important osmolyte that reduces the ROS by stimulating the activity of catalase (CAT), peroxidase (POD), superoxide dismutase (SOD), and other different antioxidant enzymes (Bogati and Walczak, 2022). Proline has an appreciable ability to bind and hydrate enzymes thereby it stabilizes and protects the macro-molecules and maintains their structural integrity and their functioning under DS (Yang et al., 2021).

**TABLE 1:** Effect of drought stress on growth, physiological, and biochemical response of plants.

Plant species	Drought stress	Effects	References
Faba bean	40% FC	DS decreases the chlorophyll contents, soluble sugars, APX, CAT, SOD, and increased the MDA and H_2_O_2_ accumulation	Kenawy et al. (2022)
Cotton	60% FC	DS decreased membrane stability, RWC, chlorophyll contents, yield components, fiber quality, and increased antioxidant activities, electrolyte leakage, phenolic, and proline contents	Eid et al. (2022)
Chinese fir	50% FC	DS reduced the RWC, root and shoot growth, chlorophyll synthesis, chlorophyll fluorescence, stomata conductance, Fv/Fm, and starch contents	Zhao et al. (2021)
Maize	DS was imposed by skipping irrigation at reproductive stage	DS decreased the plant height, cob diameter, RWC, grain and biomass, and harvest index	Mehmood et al. (2021)
Maize	20–25 % FC	DS reduced the biomass production, root shoot ratio, chlorophyll contents, stomata conductance, photosynthetic and transpiration rate, WUE, and increases, APX and SOD activity and accumulation of total soluble protein and proline	Bian et al. (2021)
Brassica	DS was imposed by skipping irrigation from flowering stage	DS reduced the chlorophyll contents, RWC, stomata conductance, grain yield and increased the proline contents	Shafighi et al. (2021)
Wheat	20% FC	DS reduced the time to heading, anthesis and maturity, RWC, chlorophyll contents, canopy temperature, assimilations production, grain yield and increase proline accumulation and oxidative stress	Chowdhury et al. (2021)
Wheat	DS was imposed by skipping irrigation from heading to grain filling stages	DS reduced the grain filling period, yield traits, grain weight, biomass yield, and harvest index of wheat	Pour et al. (2020)
Barley	10% FC	DS decreased the chlorophyll contents Fv/Fm ratio, WUE, plant height, tillers grain weight, and grain yield	Istanbuli et al. (2020)

*FC, field capacity.*

Glycine betaine (GB) is important osmolyte that plays a crucial role in plant responses against DS (Bogati and Walczak, 2022). It improves growth, photosynthetic apparatus, and anti-oxidant activities and it also protects key enzymes of the dicarboxylic acid cycle and photosystem, which play an imperious role in proper photosynthesis and respiration in plants (Ma et al., 2007). Plants under DS also synthesized different proteins including late embryogenesis abundant proteins, protein kinases, phospholipase C, phospholipase D, and G proteins, which play a significant role against DS (Bogati and Walczak, 2022). These proteins protect the functioning of ion channels, scavenge ROS, improve anti-oxidant activities, gene expression, membrane integrity, and water transportation at the cellular and sub-cellular levels and therefore improve plant tolerance against DS (Yang et al., 2021). Plants also accumulate different hormones and amino acids that play an important role in stomata regulation, osmolytes accumulation, and scavenging of ROS (Bowne et al., 2012; You et al., 2019).

## Effect of Drought Stress on Growth and Diversity of Arbuscular Mycorrhizal Fungi

The diversity of AMF species largely depends on the application and mode of the new methodology (Bahadur et al., 2019a). Around 250 AMF species have been recognized in different ecosystems (Opik et al., 2013). AMF improves plant responses against the DS which in turn improves growth and final productivity (Basu et al., 2018; Ouledali et al., 2018). Conversely, DS also has a direct impact on AMF, and water shortage significantly reduces the germination of AMF spores, AMF growth, colonization, and elongation of AMF extra-radical hyphae (Zhang et al., 2018). Over the years the major focus of researchers is to understand how DS affects the diversity as well as the composition of AMF. Generally, the behavior of AMF is opportunistic and they use their energy to produce more descendants. In addition, AMF also develop many promising characteristics to perform better in water deficit conditions (Sykorova et al., 2007). Many authors noted that *Glomus* AMF species are considered typical species of semi-arid conditions and they have an appreciable ability to grow well under DS conditions (Verma et al., 2008; Tian et al., 2009).

## The Association of Host Plant and Arbuscular Mycorrhizal Fungi Under Drought Stress

Plants use different strategies to avoid the deleterious impacts of DS. AMF modifies plant root traits which in turn increase the water uptake and reduce water loss (Bahadur et al., 2019b). Interestingly, upon exposure to DS most plants quickly ask the AMF to help them by secreting a rhizosphere molecule that is known as strigolactone (Oldroyd and Speak, 2013). In recent years AMF have gained attention globally to reduce the adverse impacts of DS (Kumar and Verma, 2018). AMF association improves plant water status at the whole plant level as indicated by high leaf relative water contents (LRWC; Barros et al., 2018). AMF association with plants under DS significantly improves plant growth by improving water and nutrient water (Kapoor et al., 2013; Pavithra and Yapa, 2018). AMF forms an extensive hyphal network which ensures better nutrient as well as water uptake by plant roots (Gong et al., 2012). Despite this AMF also affect diverse plant mechanisms including root architecture, root hydraulic conductivity, and plant photosynthetic rate (Lee et al., 2012).

Arbuscular mycorrhizal fungi-mediated response against DS involves different mechanisms including the activation of genes and metabolic pathways (Fiorilli et al., 2022). AMF improves water uptake by the host plant by triggering hormonal signaling and increasing the accumulation of osmolytes (Diagne et al., 2020). Plants tolerate drought-induced osmotic stress by increasing the accumulation of sugars, proline, and GB (Latef et al., 2016). These metabolites reduce the osmotic potential and leaf water potential (Wu et al., 2013) which allows the AMF inoculated plants to maintain higher turgor pressure which in turn improves the physiological activities particularly linked with photosynthetic apparatus (Smith et al., 2010). AMF plants also counter the drought-induced oxidative stress by scavenging ROS through enhanced anti-oxidant activities (Begum et al., 2019a). AMF also increases root growth and root hydraulic characteristics and consequently increases root’s ability to uptake more nutrients and water (Ouledali et al., 2018). Moreover, AMF hyphae also establish some beneficial pathways in soil for better nutrient acquisition and transportation and leading to a significant increase in plant growth under DS. AMF also activates plant molecular responses including activation of genes (aquaporins membrane transporters), ions, and sugar transporters (Bahadur et al., 2019a). Moreover, AMF substantially improves nutrient and water uptake which improves drought tolerance by affecting different physiological and biochemical processes (Diagne et al., 2020). Lastly, AMF also improves the plant DS tolerance through secondary responses such as improving soil stability and water holding capacity (Bahadur et al., 2019b; Hamedani et al., 2022).

## The Role of Arbuscular Mycorrhizal Fungi in Plants Under Drought Stress

Drought stress imposes serious threats to crop productivity and global food security. In recent years the role of AMF under DS is well explored. AMF protects the plants against DS by mediating biochemical, morphological, and physiological mechanisms.

## Arbuscular Mycorrhizal Fungi Maintains Membrane Stability and Plant Water Relationships Against Drought Stress

Drought stress impedes plant growth by damaging the membrane integrity (Hasanuzzaman et al., 2013); nonetheless, AMF effectively improves the membrane stability and improves the plant performance under DS (Mirshad and Puthur, 2016). Malondialdehyde (MDA) is an important indicator of membrane damage (Hassan et al., 2020) and AMF can effectively reduce MDA by 30–50% by increasing the activities of anti-oxidants (CAT and SOD), and therefore maintain the membrane integrity under DS (Li et al., 2019). AMF improves Pro, GB, and soluble sugar accumulation which protects plant proteins and membranes from under DS (Hashem et al., 2016a; Begum et al., 2019a). The mycorrhizal association also maintains higher water status in host plants which in turn maintains plant functioning under DS (Barros et al., 2018). AMF association improves root hydraulic conductivity which improves water uptake and maintains higher plant water status under DS (Augé et al., 2008). The large root surface area in the AMF association increases the water exploration area in soil which has a direct impact on LRWC, water potential, photosynthetic and transpiration rates, and crop yield (Meddich et al., 2015). Living hyphae involved in water transportation have a smaller diameter of 2–5 μm which allows them to penetrate soils that are inaccessible to root hairs and thereby absorb water and maintain higher water status in AMF inoculated plants (Allen, 2009).

Arbuscular mycorrhizal fungi also showed beneficial impacts on soil aggregate stability owing to the production of a glycoprotein known as glomalin (Wu et al., 2008) which ensures better water uptake and maintains higher plant water status (Augé et al., 2007). The glomalin in AMF association maintains soil structure stability and improves the water holding capacity which in turn maintains higher plant water content under DS (Santander et al., 2017; Gupta, 2020). AMF also regulate gene (TFT1-TFT12) expression involved in the ABA signaling pathway and improve the water status of plants in DS (Xu et al., 2018). Besides this AMF also improves plant root growth which allows plants to take more nutrient and water and resultantly maintains higher RWC under DS (Hashem et al., 2018). In conclusion, AMF regulates antioxidant activities, and gene expression, therefore maintaining membrane stability under DS.

## Arbuscular Mycorrhizal Fungi Maintains Water Use Efficiency and Nutrient Uptake Under Drought Stress

**FIGURE 2:**
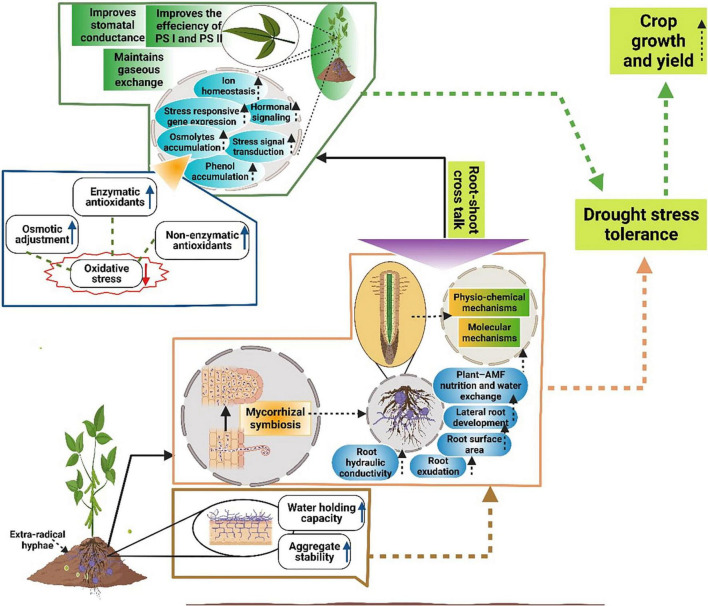
Schematic representation of different mechanisms mediated by AMF to improve growth and yield under DS. AMF improves soil aggregate stability, soil water holding capacity, root hydraulic conductance, water uptake, plant water status, root exudation, root surface area, root development, uptake of nutrients and water and leads to a significant increase in DS tolerance. Moreover, AMF improves hormonal crosstalk, osmolytes accumulations and induces signaling transduction, phenol accumulation, maintains ionic homeostasis, the efficiency of PS-I, PS-II, intercellular CO_2_ concentration resultantly improves the DS and plant growth and yield under DS.

Stomata conductance plays an important role in the photosynthetic process. DS significantly reduces the stomata conductance (Hassan et al., 2020), however, AMF improves stomata conductance resultantly improves the plant WUE (Ruíz-Sánchez et al., 2011). AMF improve gene expression coding for aquaporins and AMF mediated increase in gene expression linked with aquaporins improve water absorption (Figure 2) by plants and ensures a higher WUE under DS (Porcel et al., 2005). In some other plants like *Poncirus trifoliata* and *Rosmarinus officinalis* AMF has substantially improved the stomata conductance and plant WUE under DS (Ruíz-Sánchez et al., 2011). AMF also modify the accumulation of different hormones including ABA, jasmonic acid (JA), and strigolactones which maintain the higher LRWC and plant WUE under DS (Fernández-Lizarazo and Moreno-Fonseca, 2016). The better water uptake and higher WUE in AMF association are also linked with better root activity and higher hydraulic conductance of roots (Avio et al., 2006). In addition, increasing the ABA level works as an anti-transpirant, which reduces water loss by stomata closing thus maintaining higher WUE under DS (Mohanta et al., 2017; Egamberdieva et al., 2018). AMF also produce glomalin secretions which assist in nutrient and water uptake and lead to a significant increase in WUE under DS (Gong et al., 2012). Likewise, AMF colonization also improves root growth and root hydraulic properties and consequently maintains higher water uptake and WUE in plants facing DS (Ouledali et al., 2018). AMF hyphae also establish beneficial pathways in soils for better nutrient and water uptake which leads to a substantial increase in WUE under DS (Hamedani et al., 2022).

Arbuscular mycorrhizal fungi also maintains better nutrient uptake under DS and ensures better plant performance under DS. AMF improves calcium (Ca^2+^) uptake and Ca^2+^ is considered to play an imperative role in plant stress signaling system to attain drought tolerance (Abd_Allah et al., 2017). Potassium (K^+^) also plays an important role in the activation of various enzymes and it also up-regulates the anti-oxidant activities and osmolytes accumulation (Hashem et al., 2016b). AMF inoculation improves the plant growth under DS by increasing nitrogen (N), phosphorus (P), potassium (K), and magnesium (Mg) uptake (Egamberdieva et al., 2017). AMF inoculated plants have better root growth which maintains the higher nutrient uptake and ensures better growth under DS (Egamberdieva et al., 2017). DS impedes nutrient uptake by making nutrients inaccessible to plants owing to a reduction in roots exploration capacity in dried soils (Hameed et al., 2014). AMF improves the growth of plant roots and their exploration ability, therefore, maintains better nutrient uptake in AMF inoculated plants under DS (Bowles et al., 2018). AMF hyphae can also explore the soil pores which plant roots cannot explore, thereby, AMF maintains better nutrient uptake in plants under DS (Zhao et al., 2015). In another study, it was noted that AMF application appreciably improved the N, P, and Mg uptake and ensured better nutrient uptake for plants growing under DS (Miransari et al., 2007).

Arbuscular mycorrhizal fungi makes better assimilation of N in plants by increasing the activity of nitrate reductase (NR) enzymes (Garg, 2013). An increase in N status in mycorrhizal is due to increase transportation of N through AMF hyphae which also increases the concentration of P which is needed for the phosphorylation of NR where there is a low concentration of N (Caravaca et al., 2003). Phosphatases enzyme plays an important role in the absorption, assimilation, and metabolism of P (Abd_Allah et al., 2015). AMF improves phosphatase activity, which increases the organically bound P and therefore makes it available for uptake and transport (Egamberdieva et al., 2017). In conclusion, AMF increases gene expression is linked with aquaporins that improve water absorption and ensures a higher WUE under DS. Moreover, an increase in nutrients owing to AMF substantially improves the plant growth and development under DS.

## Arbuscular Mycorrhizal Fungi Maintains Plant Photosynthetic Performance Under Drought Stress

Drought stress negatively affects photosynthesis and causes a significant reduction in assimilate production for plants. DS reduces photosynthesis by decreasing the chlorophyll contents and increasing the production of ROS (Sochacki et al., 2013). AMF effectively improve chlorophyll synthesis and maintains better photosynthesis and subsequent assimilate production by reducing the ROS formation (Hashem et al., 2016a). DS also reduces photosynthesis by reducing adenosine triphosphate (ATP) production, stomata, and non-stomata limitations (Hassan et al., 2020). However, AMF significantly improved the gas exchange characteristics and fluorescence parameters, nutrient and water uptake, and RuBisCO activities, therefore, ensuring better photosynthesis under DS (Begum et al., 2019b).

Drought stress triggers the reduction in photosynthesis by reducing RuBisCO synthesis and up-regulating the activity of chlorophyll degrading enzyme (chlorophyllase) (Dalal and Tripathy, 2012; Fatima et al., 2014). However, AMF substantially increased the chlorophyll contents and maintain better synthesis of RuBisCO and leading to a significant increase in photosynthetic rate under DS (Abdel-Salam et al., 2018). AMF also increased photosynthesis by affecting both stomatal and non-stomatal parameters. Likewise, AMF improved stomata conductance which increases the entry of CO_2_ into plant leaf tissues which in turn increases the efficiency of PS-II resultantly improving photosynthetic efficiency under DS (Zhou et al., 2015; Mo et al., 2016). Drought stress down-regulated the enzymes responsible for the synthesis of chlorophyll, however, at the same time DS increases the activity of chlorophyll degrading enzyme (chlorophyllase) (Zhu et al., 2017). However, AMF substantially decreases the activity of chlorophyllase and maintains the activity of genes and enzymes involved in chlorophyll synthesis thereby maintaining higher chlorophyll synthesis under DS (Hashem et al., 2016b; Alwhibi et al., 2017; Zhu et al., 2017). In another study, it was noted that AMF inoculation improved the chlorophyll contents, efficiency of PS-II, and root hydraulic conductivity (Calvo-Polanco et al., 2016). AMF inoculation also improved the carotenoid contents which protect photosynthetic apparatus from drought-induced oxidative stress and ensured optimum photosynthesis under DS (Abd Allah et al., 2015).

It is a well-known fact that plants facing the DS show a significant reduction in photosynthesis owing to ROS that damage the photosynthetic apparatus which can reduce/limit the supply of ATP and reduce nicotinamide adenine dinucleotide phosphate (NADPH) to the Calvin cycle (Abbaspour et al., 2012). However, AMF symbiosis improves plant water status which increases stomata conductance and therefore increases CO_2_ fixing and improves the ATP and NADPH supply to Calvin supply and resulting in better photosynthesis and assimilate production (Boldt et al., 2011; Estrada et al., 2013). DS also decreases the efficiency of PS-II and Fv/Fm ratio, and it has been reported that AMF shows a positive effect on PS-II efficiency which in turn improves the plant photosynthesis under DS (Sánchez-Blanco et al., 2004; Manoharan et al., 2010). To summarize, AMF improves antioxidant activities which protect the photosynthetic apparatus, therefore maintaining higher photosynthetic efficiency under DS.

## Arbuscular Mycorrhizal Fungi Maintains Osmolyte Accumulation and Confers Drought Tolerance

Osmolyte accumulation is an important strategy used by plants to cope with the deleterious impacts of DS. AMF inoculation significantly increased the accumulation of sugars, proline, and GB, which confer DS tolerance in plants. DS also triggers the synthesis of glucose and proline, however, AMF further increased the synthesis of these osmolytes and improves the drought tolerance (Mona et al., 2017; Wu et al., 2017). AMF is considered to upregulate the synthesis of enzymes involved in proline synthesis and AMF mediated increase in proline accumulation protects proteins (Table 2) and membranes from ROS (Abd Allah et al., 2015; Hashem et al., 2015). The accumulation of proline in plants also affects the metabolism of N which is needed for stress recovery. AMF significantly improves the activity of N metabolizing enzymes resulting in a marked increase in the accumulation of energy-rich amino acids (Hameed et al., 2014) which enhanced the drought tolerance (Doubková et al., 2013; Ouledali et al., 2018) and biomass production in plants grown under DS (Zou et al., 2013).

**TABLE 2:** Effect of AMF on growth and physiological traits on different plants grown under DS.

Plant species	Drought stress	Effects	References
Soybean	40% FC	AMF improved the RWC, proline contents, chlorophyll contents, pods, grain weight, grain, and biomass	Igiehon et al. (2021)
Rice	50% FC	AMF improved the RWC, plant height, chlorophyll contents, panicles per plant, root and shoot dry matter, grain weight, grain yield, and WUE	Das et al. (2021)
Soybean	DS was imposed by skipping irrigation and pod and seed development stage	AMF improved the plant biomass, chlorophyll contents, IAA contents, branches per plant, nodules/plant, and grain yield	Sheteiwy et al. (2021)
Tobacco	30% FC	AMF improved leaves/plant, plant height, plant biomass, chlorophyll contents, Fv/Fm, free amino acids, proline, total phenols, and essential oils	Begum et al. (2021)
Sorghum	DS was imposed by skipping irrigation	AMF inoculation improved chlorophyll contents, N contents, proline contents, panicles per plant, and 1000 grain weight	Kamali and Mehraban (2020a)
Wheat	22% FC	AMF inoculation improved the RWC, chlorophyll contents, chlorophyll fluorescence, efficiency of PS-I and PS-II	Mathur et al. (2019)
Strawberry	35% FC	AMF improved the root and shoot biomass, RWC, stomata conductance, photosynthetic rate, WUE, free amino acids, soluble sugars, proline, and uptake of Mn, Fe, Si, and Zn	Moradtalab et al. (2019)
Maize	40% FC	AMF inoculation increased the root and shoots growth, N, P, K, Ca, and Mg uptake and WUE of maize plants	Zhao et al. (2015)
Soybean	55% FC	AMF increased the plant height, root length, root surface area and soluble sugars	Liu et al. (2013)

Arbuscular mycorrhizal fungi inoculation also increases the accumulation of nitrogenous compounds and free polyamines in water-deficient plants (Rapparini and Peñuelas, 2014). The increase in the accumulation of polyamines adjusts the plant osmotic potential in DS which is considered to be an important protective mechanism of AMF against DS (Bakr et al., 2018). Some authors noted that AMF decreased the accumulation of soluble sugars and improve the plant tolerance against DS (Zhang et al., 2010). Contrarily, some authors also noted a positive correlation between AMF and accumulation of soluble sugars which might be due to the sink effect on the AMF that demands sugar from plant shoot tissues (Yooyongwech et al., 2013). AMF also caused a substantial increase in the accretion of free amino acids (FAA) to increase NR activity which contributes toward to a great accumulation of FAA which in turn improve tolerance against DS (Zarea et al., 2011; Ahanger et al., 2017). Improved GB accumulation protects the photosynthesis and RuBisCO activity and plants from the damaging effects of DS (Khan and Hakeem, 2014). Drought tolerance in plants is upregulated by anti-oxidant activities which are further enhanced by the accumulation of GB and proline (Hashem et al., 2016a). Additionally, AMF-mediated increase in osmolytes accumulation improved the functioning of RuBisCO, and protects the photosynthetic apparatus from ROS which in turn improved the plant performance under DS (Mo et al., 2016).

## Arbuscular Mycorrhizal Fungi Maintains Hormonal Crosstalk to Confer Drought Tolerance

Drought stress causes a significant increase in ABA bio-synthesis which increases the ABA level in plants and induces the stomata closure which minimizes the water loss by transpiration (Pozo et al., 2015; Xie et al., 2018). ABA is an abiotic stress hormone and a reduction in ABA level may explain why an AMF-associated plant has more tolerance against DS. AMF are considered to be indispensable for sustaining the AMF colonization, especially in unfavorable conditions such as drought conditions (Ludwig-Müller, 2010). ABA improves AMF colonization, functionality as well as development (Herrera-Medina et al., 2007; Aroca et al., 2013). Strigolactones are newly discovered plant hormones that regulate plant architecture and reproductive development (Foo and Reid, 2013). However, they were initially identified for their intermediation capacity in the AMF symbiosis, where they work as a signaling molecule for plants under unfavorable conditions (López et al., 2010).

Arbuscular mycorrhizal fungi colonization in the host plant also activates the jasmonic acid (JA) signaling pathway (Tejeda et al., 2008; Asensio et al., 2012). Jasmonic acid together with ABA plays an important role development and functionality of AMF. Auxins (IAA) and gibberellic acid (GA) play an imperious role in the growth of plants under stress conditions (Vishwakarma et al., 2017). AMF colonization significantly increases the accumulation of IAA, GA, and JA which improved the plant performance under stress and drought tolerance (Egamberdieva et al., 2018; Hashem et al., 2018; Sánchez et al., 2018). Drought tolerance in plants is up-regulated by anti-oxidant activities which are further enhanced by the accumulation of GB and proline (Hashem et al., 2016a). To summarize, AMF inoculation improves the synthesis and accumulation of osmolytes and hormones which maintains better plant performance under DS.

## Arbuscular Mycorrhizal Fungi Improves the Accumulation of Phenols and Enzyme Activity to Confer Drought Stress

Phenolic compounds have excellent anti-oxidant activity and they play a significant role in stress conditions. AMF significantly increases the accumulation of phenolic substances, which strengthens the anti-oxidant defense system and improves the tolerance against DS (Begum et al., 2019a). Polyphenolic substances are active scavengers of free radicals and protect cell structures and their functioning (Hazzoumi et al., 2015). AMF substantially increase the accumulation of phenolic compounds by 50–60% which substantially improves the DS tolerance (Amiri et al., 2015; Kasote et al., 2015). AMF inoculation increases the accumulation of phenols and flavonoids which play an important in mycorrhization, adaptation, and growth of plants by inducing cellular signaling (Steinkellner et al., 2007; Mandal et al., 2010).

Phenylalanine ammonia-lyase (PAL) is found abundantly in higher plants that play a crucial role in plant metabolism and this enzyme also improves plant protection against biotic and abiotic stresses (Osakabe et al., 2007). AMF inoculation substantially improved the up-regulation of PAL which in turn improved the plant growth and provide protection against DS (Osakabe et al., 2007; Hao et al., 2016; Datrindade et al., 2019). Further AMF also increases the concentration of total GRSP (T-GRSP), easily extractable (EE-GRSP), and difficultly extractable (DE-GRSP) under DS. Glomalin-related soil proteins (GRSP) contain different cations, carbohydrates, proteins, and aliphatic components and work as glue to bind soil particles (Rillig et al., 2001). AMF substantially increases T-GRSP, EE-GRSP, and DE-GRSP under DS and ensures better water uptake and plant tolerance against DS (Begum et al., 2021).

## Arbuscular Mycorrhizal Fungi Strengthens the Anti-Oxidant Defense System to Confer Drought Tolerance

The major effect of DS is the production of ROS that damage plant cellular membranes, proteins, and lipids. Nonetheless, AMF improve anti-oxidant activities and mitigate the drought-induced ROS effects on plants (Egamberdieva et al., 2017). Stress conditions up-surge the ROS which causes lipid-peroxidation and affects the functioning and fluidity of cellular membranes. However, AMF significantly increases the activity of antioxidants [superoxide dismutase (SOD), catalase (CAT), ascorbate peroxidase (APX), glutathione reductase (GR), ascorbic acid (AsA), and glutathione synthetase (GSH)] which in turn increases the cellular stability (Hashem et al., 2016a,b). Superoxide dismutase is the first line of defense against oxidative stress and APX, GR, AsA, and GSH (Table 3) are key components of ROS scavenging pathways. AMF increases the activity of the aforementioned antioxidant and neutralizes H_2_O_2_ by preventing the formation of toxic OH and protecting plant mitochondria and electron transport (Abd_Allah et al., 2015; Mona et al., 2017). The better maintenance of AsA-GSH components ensures NADP availability in order to keep electron transport at a normal rate. AMF substantially improves the activities of AsA and GSH which in turn maintain NADP availability and electron transport thus improving photosynthesis under DS (Scheibe and Dietz, 2012; Abd Allah et al., 2015). Many other authors also reported that AMF significantly increases the APX, CAT, SOD, and GR activities which demonstrated greater protection of the photosynthetic apparatus and subsequently improve plant growth (Mirzai et al., 2013; Weng et al., 2015; Yang et al., 2015).

**TABLE 3:** Effect of AMF on oxidative stress markers, antioxidant activities and genes expression under DS.

Plant species	Drought stress	Effects	References
Wheat	50% FC	AMD reduced the ROS and improved the activities of CAT, APX, GR, and SOD	Tereucán et al. (2022)
Tobacco	30% FC	AMF reduced MDA contents and improved APX, CAT, POD, SOD, GSH, and AsA activities	Begum et al. (2021)
White clover	55% FC	AMD decreased the MDA accumulation and significantly increased the CAT, POD, and SOD activities	Liang et al. (2021)
Date Palm	25% FC	AMF improved membrane stability, reduced EL, and increase activities of CAT, POD, SOD and GSH	Harkousse et al. (2021)
Soybean	DS was imposed by skipping irrigation and pod and seed development stage	AMF reduced the ROS, MDA accumulation and increased activities of POD, CAT, and expression of P5CS, P5CR, PDH, P5CDH, GmSPS1, GmSuSy, and GmC-INV	Sheteiwy et al. (2021)
Orange	55% FC	AMF improved the expression of PtMn-SOD, PtCAT1, and PtPOD and activities of SOD and CAT and reduced the EL, MDA, and H_2_O_2_ accumulation	He et al. (2022)
Sorghum	DS was imposed by skipping irrigation	AMF reduced the MDA accumulation and EL and increased the activities APX, POD, and CAT	Kamali and Mehraban (2020b)
Tea	55% FC	AMD significantly decreased the MDA accumulation, and activities of CAT and SOD and expression of CsCAT and CsSOD genes	Liu et al. (2020)
French bean	DS was imposed by withholding irrigation for 5 days	AMF reduced the MDA contents increase activities of CAT, POD, SOD, and GSH	Prabha and Sharadamma (2019)
Ryegrass	25% FC	AMF significant reduced the MDA accumulation, EL and increased the activities of CAT, POD, and SOD	Yang et al. (2019)

*FC, field capacity.*

Likewise, an increase in AsA and glutathione (GT) has been reported in *Brassica napus* and *Sesbania sesban* plants inoculated with AMF resulting in greater stress tolerance (Shafiq et al., 2014; Abd Allah et al., 2015). The *Brassica napus* are considered to fall among the plants that do not form arbuscules within the roots, however, beneficial impacts of AMF association can be attained without a complete mycorrhizal association (Fernandez et al., 2020). SOD induced dis-mutation of superoxide radicals produced in plant chloroplast prevent damage to photosynthetic apparatus (Per et al., 2016). AMF-mediated increase in SOD activity prevents the production of hydroxyl radicals through the Haber–Weiss reaction, which can otherwise pose serious damage to plant membranes and organelle functioning (Ahanger et al., 2017). Likewise, AMF-mediated increases in CAT and POD neutralize excessive H_2_O_2_ in cytosol whereas APX, GR, AsA, and GSH work as an intriguing pathway for neutralizing the H_2_O_2_ in plant chloroplast and mitochondria (Ahanger et al., 2017). Many other authors also demonstrated that AMF inoculation upregulates anti-oxidant activities, increases photosynthesis, and maintains the redox homeostasis in plants grown under DS (Yang et al., 2015; Mo et al., 2016). AsA and GSH are key components of redox buffer and they can also scavenge ROS directly and AMF up-regulates these anti-oxidants to counter the stress conditions (Weng et al., 2015; Begum et al., 2019b). In conclusion, the AMF-mediated increase in antioxidant activities alleviates drought-induced toxic effects and maintains better plant performance.

## Arbuscular Mycorrhizal Fungi Regulates Gene Expression Aquaporins Activity to Confer Drought Stress Tolerance

Aquaporins are involved in the transportation of water in plant roots and shoots. They also play an important role in plant growth, fixation of CO_2_, nutrient allocation, and plant interactions with different abiotic stresses (Maurel et al., 2015). Recently, AQP functions in plant-water relations received considerable attention as an important way to improve crop performance (Moshelion et al., 2015; Tharanya et al., 2018). AQP maintains the root water balance with transpiration (Sadok and Sinclair, 2010) by increasing root hydraulic conductivity upon high transpiration demand (Sakurai et al., 2011). In some crops, AQP functioning is also improved under lower transpiration which is also considered to be beneficial for crop yields (Kholová et al., 2012). AQP is considered to be involved in diverse physiological mechanisms that determine the pattern as well as the rate of plant water usage (Vadez et al., 2013). Further AMF also enhanced the root hydraulic conductance and maintains hydraulic continuity between plant roots thereby reducing the drop in matric potential at the root-soil furnace and enhancing the water uptake by AMA inoculated plants (Quiroga et al., 2019a; Pauwels et al., 2020; Abdalla and Ahmed, 2021).

Arbuscular mycorrhizal fungi symbiosis activates different molecular mechanisms including activation of gene expression, aquaporins (AQP) membrane transporters, sugar, and ion transporters to cope with the impacts of DS (Bahadur et al., 2019a). The AMF-mediated increase in expression of AQP improves nutrient and water uptake and mitigates the drought-induced toxic effects (Bahadur et al., 2019a). Likewise, in tomato (*Solanum lycopersicum*) plants AMF increased the DS by regulating the expression of genes (TFT1-TFT12) involved in the ABA signaling pathway (Xu et al., 2018). The AMF-mediated increase in D-myo-inositol-3-phosphate synthase (IPS) and 14-3-3-like protein GF14 (14-3GF) expression improved the plant tolerance against DS (Li et al., 2016). In another study, it was noted that expression of two AQP genes (GintAQPF1 and GintAQPF2) was markedly increased in mycorrhizal roots in response to DS which supports the evidence of direct AMF involvement in improving the plant tolerance against DS (Li et al., 2013). Consistently, another group of authors also noted a significant increase in AQPs gene expression in AMF-inoculated roots cells under DS which in turn increased the tolerance against DS by increasing nutrient and water uptake and better architecture of the root system (Neumann et al., 2009; Zou et al., 2015). AMF symbiosis clearly regulates AQP expression and improves root hydraulic conductivity, root water status, and tolerance against DS (Aroca et al., 2012; Bárzana et al., 2014).

The increase in expression of AQPs in AMF inoculated plants substantially increased the root hydraulic conductivity which in turn increased plant performance under DS (Sanchez-Romera et al., 2016). In another study, it was noted that AMF increases the expression of PtAHA2 genes in plant leaves and roots under DS and leads to a significant increase in photosynthetic and transpiration rates, intercellular CO_2_ concentration, and stomata conductance (Cheng et al., 2021). TdSHN1 is considered to be involved in plant tolerance against different abiotic Djemal and Khoudi (2015) AMF considerably increased the expression of SHN1 which in turn improved the plant WUE by modifying the leaf diffusive characteristics owing to the accumulation of high levels of wax (Aharoni et al., 2004; Fiorilli et al., 2022). Another gene named dehydration responsive element binding protein (DREB) has been reported to play an important role against DS (Latini et al., 2013). In a study, it was noted AMF improve the expression of TdDRF1 and induce drought tolerance in wheat plants (Fiorilli et al., 2022). AMF also up-regulates the expression of MdGH3-2 and MdGH3-12 which improve the plant RWC, photosynthetic capacity, chlorophyll contents, and scavenging of ROS in apple plants (Huang et al., 2021). Conversely, silencing of MdGH3-2/12 genes from apple plants negatively affected AMF colonization, plant growth, and subsequent development under DS (Huang et al., 2021). AMF increase the genes’ expression which in turn increased the tolerance against DS by increasing nutrient and water uptake and better architecture of the root system.

## Arbuscular Mycorrhizal Fungi Improves Growth, Yield, and Quality Under Drought Stress

Arbuscular mycorrhizal fungi colonization significantly improves plant growth and development under DS (Table 2). AMF-mediated increases in growth under stress can be attributed to an increase in the accumulation of growth-promoting hormones, better nutrient and water uptake, and scavenging of ROS (Egamberdieva et al., 2017; Vishwakarma et al., 2017). AMF also maintain optimum nutrient availability and plant tissue water status which in turn improves the overall performance of plants under DS (Abdel-Salam et al., 2018). AMF also modifies root characteristics including root diameter, root morphology, and promotes a dense root system which allows better nutrient (N, P, and K) and water uptake and resultantly ensures better plant growth under DS (Chitarra et al., 2016; Begum et al., 2019b). Moreover, AMF inoculation also increases the plant height, leaves per plant, and biomass production by improving P uptake, water acquisition, and cellular signaling in plants under DS (Li et al., 2013; Jayne and Quigley, 2014; Begum et al., 2021). In another study, Sheteiwy et al. (2021) noted that DS significantly reduces the grain yield of plants. However, AMF (*Bradyrhizobium*) inoculation considerably increased the growth and yield by decreasing lipid peroxidation through enhanced antioxidant activities (CAT and POD) and accumulation of proline under DS (Sheteiwy et al., 2021).

Arbuscular mycorrhizal fungi also improve the plant metabolism, which affects both the quality and quantity of secondary metabolites quality (Karagiannidis et al., 2011; Fokom et al., 2019). Likewise, in tobacco plants, AMF improved the accumulation of essential oil (EO) which was linked with a change in the secondary metabolism of the plant (Begum et al., 2021). In another study, it was reported that AMF in combination with vermicompost significantly improved plant height, root and shoot length, biomass, and grain yield of quinoa plants under well water and DS (Cozzolino et al., 2016; Lahbouki et al., 2022). Likely, the beneficial impacts of AMF in improving the plants growth are also linked with improved nutrient cycling, better nutrient and water uptake, bio-degradation of organic matter, hormone production, and improvement in soil properties (Cavagnaro, 2015; Zhang et al., 2020; Benaffari et al., 2022).

## Implications of Arbuscular Mycorrhizal Fungi for Improving Nitrogen Use Efficiency

**FIGURE 3:**
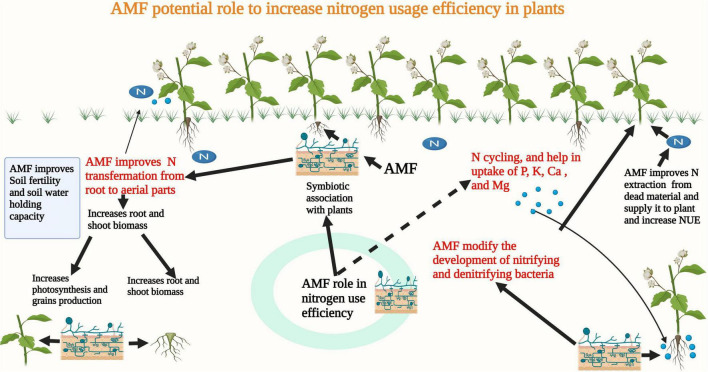
AMF forms a symbiotic association with plants and it improves N extraction from dead materials and it also improves microbial activities and N uptake, thereby improving NUE in plants.

Nitrogen (N) is an essential nutrient needed for plant growth and development. However, its deficiency in agricultural soils is a major problem. Therefore, to fulfill plant nutrient needs farmers use a large quantity of N fertilizers, which is also posing a serious threat to the environment (Varinderpal-Singh et al., 2020). The poor recovery efficiency of N fertilizers poses serious economical, environmental, and ecological losses (Varinderpal-Singh et al., 2020). Inappropriate timing and excessive use of N fertilizers are major reasons for the lower recovery efficiency of N fertilizers (Varinderpal et al., 2021). Globally, different strategies are being used to improve the efficiency of applied nutrients. Among these, the use of microbes is considered an important and effective approach to improve N use efficiency. Many authors have reported the role of AMF in the uptake of nutrients (Figure 3) which can appreciably improve the growth of the host plant (Smith et al., 2011). The formation of a hyphal network in AMF inoculated plants limits the inefficient use of applied N fertilizers. AMF play an imperative part in N cycling by altering microbial compositions and modifying the development of denitrifying, nitrifying, and free-living bacteria (Veresoglou et al., 2011).

In many crops, N is considered to be a limiting factor and many authors have noted that AMF absorbs and transfers N to nearby plants and improves their growth and development (Battini et al., 2017; Turrini et al., 2018). In another study, Zhang et al. (2018b) noted that AMF increases the shoot biomass and panicles and grains production by increasing the N uptake and its redistribution to plants. AMF form a symbiotic relationship that improves nutrient uptake particular N which in turn improves the plant growth and development (Battini et al., 2017). The interaction between the AMF and salinity stress significantly affects the N and P concentration in plant parts. AMF significantly improve N contents and NUE in crop plants (Turrini et al., 2018). It has been reported that AMF can take a significant amount of N from dead and decomposed materials then provide N to host plants and improve N uptake and NUE of the host plant (Turrini et al., 2018).

The extra-radical hyphae of AMF absorb and assimilate inorganic N (Jin et al., 2005) and it has been reported that 20–75% N uptake by AMF is transferred to the host plant which in turn improves NUE in host plants (Hashem et al., 2018). The increase in the N uptake in AMF-inoculated plants increases chlorophyll synthesis which improves the plant’s photosynthetic efficiency and subsequent assimilate production (Deandrade et al., 2015). AMF symbiosis also improves C and N accumulation under elevated CO_2_ concentration and ambient conditions (Zhu et al., 2016). Moreover, AMF also improve the N, P, K, Ca, and Mg uptake under DS (Asrar et al., 2012; Bati et al., 2015). Additionally, AMF also proved beneficial in accumulating the Fe, Mn, Mg, and Na and protecting the plants from the toxic effects of these metals (Bati et al., 2015). A significant positive relationship was noted between N accumulation and biomass production which suggested that an increase in dry matter production was associated with an increase in N uptake in AMF inoculated plants (Zhu et al., 2016). In another study, Fellbaum et al. (2012) noted that C supply increases the N uptake and transportation in AMF symbiosis. Moreover, NUE of AMF inoculated plants is considered to be higher at elevated CO_2_ as compared to non-AMF-inoculated plants which suggested that AMF colonization may change the balance of N and C under elevated CO_2_ by changing the C demands of AMF and supply of N from AMF to host plants (Cavagnaro et al., 2011).

Arbuscular mycorrhizal fungi plays an imperative role in N cycling by absorbing N and then supplying it to host plants (Bonfante and Genre, 2010). The extraradical mycelium of AMF can directly absorb ammonium, nitrate, and amino acids and improve the N uptake and subsequently N use efficiency in plants (Basu et al., 2018). Additionally, AMF are also vital for the translocation of N and which in turn increases the NUE and neutralizes the excessive N fertilization (Leifheit-Eva et al., 2014; Rosolem et al., 2017). AMF assist plant roots to supply soil nutrients to host plants which are largely depend on symbiosis reaction between strigolactones, AMF “Myc factors” and root exudates of host plant roots (Basu, 2018) thereby this association improve the N uptake resultantly NUE in plants (Xing et al., 2019). Additionally, strigolactones also adjust root development and shoot branching (Seto et al., 2012) and significantly influence the N distribution as well as N translocation to different shoot tissues (Luo et al., 2018). Moreover, AMF forms an interconnected hyphal network which improves the N uptake and NUE in host plant (Verzeaux et al., 2017). The beneficial impact of AMF in improving N uptake and NUE has been highlighted in a meta-analysis of more than 300 field studies. The results of the meta-analysis indicated that the use of AMF is a profitable practice and has many agronomic uses and improves N uptake and subsequently NUE in plants (Mensah et al., 2015).

## Conclusion and Future Prospective

Drought stress causes a serious reduction in plant growth and development by disturbing plant physiological, biochemical, and molecular responses. However, AMF protects plants under DS and substantially improves their growth and development. AMF inoculation maintains membrane integrity and plant water status, protects photosynthetic apparatus from drought-induced oxidative stress, and improves the synthesis of photosynthetic pigments thereby improving plant growth and development under DS. AMF also improves the accumulation of osmolytes and, hormones and gene expression and anti-oxidant activities which leads to an appreciable improvement in plant performance in water deficit conditions. Besides this, AMF also improves the expression of aquaporins and improves the water uptake and water use efficiency which are major reasons for AMF-mediated improvement in plant growth under DS. Additionally, AMF also improves soil health and plays an important role in soil nutrient cycling and improves nutrient uptake and nutrient use efficiency and alleviates negative impacts of DS, and improves plant growth and yield.

Despite recent progress about the role of AMF in mitigating the adverse effects of DS, there are many unanswered questions. The role of AMF in seed germination mechanism is poorly studied, therefore it is important to determine how AMF affect the germination mechanism and improve seed germination under DS. The role of AMF in nutrient uptake is well studied by authors, however, its role in nutrient signaling, ion transport, and nutrient channels is not explored. Therefore, it would be fascinating to perform research on these aspects to increase our understanding of the role of AMF against DS. The role of AMF in improving photosynthesis is clearly explored nonetheless, its role in stomata movements is still unknown therefore, it is suggested to explore the role of AMF in stomata signaling and its impacts on anion channels in leaf guard cells under DS.

The role of AMF on hormones (ABA, GA, and IAA) and osmolytes (proline and GB) accumulation under DS is will be studied by the authors. However, it would be interesting to explore the relationship between AMF cytokinin, ethylene, salicylic acid, proline, and GB at the transcriptomic level. Also, it would also be interesting to understand the effect of AMF on genes and enzymes involved in the synthesis of these compounds under DS. The role of AMF on pollen viability and quality of crops is not explored well, therefore, it is needed to understand the role of AMF in improving pollen viability and final crop quality under DS. Likewise, the identification of host plant and AMF-specific protein factors regulating the symbiotic relationship and major cellular as well as metabolic pathways under DS can also be an important area of research. The role of AMF on nitrogen use efficiency (NUE) is well explored in plants. However, more studies are needed at the transcriptomic, proteomics, and metabolomics level to elucidate the role of AMF in N uptake and its effect on NUE. Lastly, more studies are direly needed to determine the effect of AMF on aquaporins and their role in plant water uptake under DS.

## Author Contributions

HT and MH: conceptualization and writing original draft. LF, JM, YL, MN, AS, and SQ: reviewing and editing. All authors contributed to the article and approved the submitted version.

## Conflict of Interest

The authors declare that the research was conducted in the absence of any commercial or financial relationships that could be construed as a potential conflict of interest.

## Publisher’s Note

All claims expressed in this article are solely those of the authors and do not necessarily represent those of their affiliated organizations, or those of the publisher, the editors and the reviewers. Any product that may be evaluated in this article, or claim that may be made by its manufacturer, is not guaranteed or endorsed by the publisher.
